# Ancient Nursery Area for the Extinct Giant Shark Megalodon from the Miocene of Panama

**DOI:** 10.1371/journal.pone.0010552

**Published:** 2010-05-10

**Authors:** Catalina Pimiento, Dana J. Ehret, Bruce J. MacFadden, Gordon Hubbell

**Affiliations:** 1 Department of Biology, University of Florida, Gainesville, Florida, United States of America; 2 Division of Vertebrate Paleontology, Florida Museum of Natural History, Gainesville, Florida, United States of America; 3 Center of Paleoecology and Archaeology, Smithsonian Tropical Research Institute, Panama, Republic of Panama; 4 School of Natural Resources and Environment, University of Florida, Gainesville, Florida, United States of America; 5 Division of Research on Learning (DRL), Education and Human Resources (EHR), National Science Foundation, Arlington, Virginia, United States of America; 6 Jaws International, Gainesville, Florida, United States of America; Paleontological Institute, Russian Federation

## Abstract

**Background:**

As we know from modern species, nursery areas are essential shark habitats for vulnerable young. Nurseries are typically highly productive, shallow-water habitats that are characterized by the presence of juveniles and neonates. It has been suggested that in these areas, sharks can find ample food resources and protection from predators. Based on the fossil record, we know that the extinct *Carcharocles megalodon* was the biggest shark that ever lived. Previous proposed paleo-nursery areas for this species were based on the anecdotal presence of juvenile fossil teeth accompanied by fossil marine mammals. We now present the first definitive evidence of ancient nurseries for *C. megalodon* from the late Miocene of Panama, about 10 million years ago.

**Methodology/Principal Findings:**

We collected and measured fossil shark teeth of *C. megalodon*, within the highly productive, shallow marine Gatun Formation from the Miocene of Panama. Surprisingly, and in contrast to other fossil accumulations, the majority of the teeth from Gatun are very small. Here we compare the tooth sizes from the Gatun with specimens from different, but analogous localities. In addition we calculate the total length of the individuals found in Gatun. These comparisons and estimates suggest that the small size of Gatun's *C. megalodon* is neither related to a small population of this species nor the tooth position within the jaw. Thus, the individuals from Gatun were mostly juveniles and neonates, with estimated body lengths between 2 and 10.5 meters.

**Conclusions/Significance:**

We propose that the Miocene Gatun Formation represents the first documented paleo-nursery area for *C. megalodon* from the Neotropics, and one of the few recorded in the fossil record for an extinct selachian. We therefore show that sharks have used nursery areas at least for 10 millions of years as an adaptive strategy during their life histories.

## Introduction

Sharks, especially large species, are highly mobile organisms with a complex life history and wide distribution. During their lifetime they generally utilize three types of areas: adult feeding, reproduction and nurseries [1]. In modern species, nursery areas are historically defined by the presence of gravid females and free-swimming neonates. It is also an area that can be shared by several shark species, where young sharks spend their first weeks, months or years [2]. More recent studies have defined nursery areas as geographically discrete essential zones for shark survival [3] that provides them with two types of benefits: protection from predation (mainly larger sharks [2]) and abundant food resources. Productive, shallow-water ecosystems thus provide sharks significant protection from larger predators and/or abundant food resources, both of which are essential to survival [4].

**Figure 1:**
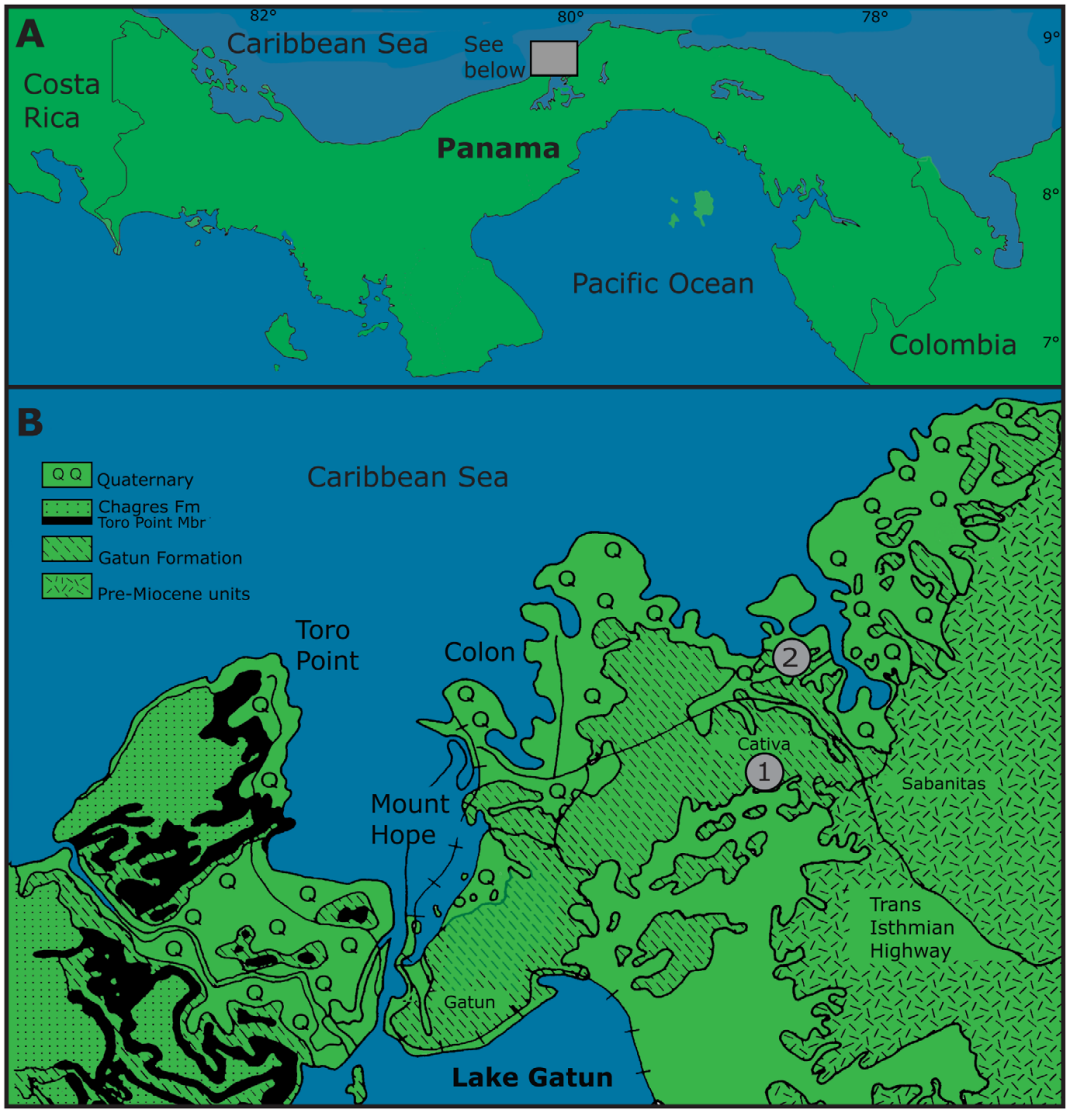
Study area. A. Location of Panama and the Gatun Formation. The shaded box represents the general study area in northern Panama. B. Expanded geological map (from “See Below” shaded box in Fig. A). This map shows the exposures of the Gatun Formation and surrounding rock units (modified from Coates et al., 1992). The two fossil localities collected from the Gatun Formation during this study include: (1) Las Lomas and (2) Isla Payardi.

The Gatun is a highly fossiliferous Neogene formation located in the Isthmus of Panama (Figure 1) with a diverse fauna of sharks [5]–[7]. It was located within a marine strait that connected the Pacific Ocean and the Caribbean Sea during the late Miocene (∼10 Ma) [8]. Studies of different taxa, including the exceedingly diverse molluscan fauna, indicate that it was a shallow-water ecosystem (∼25 m depth) with higher salinity, mean annual temperature variations, seasonality and productivity relative to modern systems in this region [7], [9]–[13]. Over the past 20 years, the Gatun Formation localities have been extensively used to extract sediment for construction. During the more recent years, these extraction activities have increased substantially. Based on our observations made during the two past years of fieldwork, we predict that these outcrops will soon likely be excavated completely. Therefore it is timely and urgent to study the fossils occurring in these outcrops before they are no longer available to science.

Fossil sharks were first reported from Panama in 1862 [5]. In 1984, the first description of the elasmobranchs from the Gatun Formation was published [6]. More recently, in 2010 the biodiversity of the fossil sharks from the Gatun has been documented from large new collections, and comprise 16 recognizable taxa. This work also included paleoecological and paleodepth analyses that supported the interpretation of the paleoecology of the Gatun Formation as shallow-water habitat in a productive environment [7].

Although it is not very common, the extinct *Carcharocles megalodon* (Agassiz 1843) is one of the species that occurs in the Gatun Formation. The taxonomic assignment of this species has been debated for nearly a century, and there are three possible interpretations: (1) Some authors place *C. megalodon* and other megatoothed sharks with the extant white shark (*Carcharodon carcharias*) in the same genus (*Carcharodon*) and therefore the same family (Lamnidae) [14]–[16]. (2) Other authors place *C. megalodon* and megatoothed sharks in a different genus (*Carcharocles*) and family (Otodontidae) [17]–[23]. Although a minority point of view, some workers recognize (3) megatoothed sharks as a series of chronospecies of the genus *Otodus*, and place all megatoothed sharks except *C*. *megalodon* in this genus. Furthermore, *C*. *megalodon* is assigned to the genus *Megaselachus*, based on the loss of lateral cusplets [24]. We follow the second hypothesis; that *Carcharocles megalodon* and *Carcharodon carcharias* belong to separate genera in different families. However, both species belong to the order Lamniformes, and in the absence of living members of the Otodontidae, *C*. *carcharias* should be regarded as ecologically analogous species to *C. megalodon*. We base this analogy on the fact that both species share similar ecological niches with presumed similarities in body shape, feeding habits, and overall tooth and vertebral centrum morphology. Even though these species are not direct relatives, no other extant lamniform species share as many characteristics with *C. megalodon* as does *C. carcharias*.

*C. megalodon* is widely regarded as the largest shark to have ever lived. Based on tooth crown height (CH), this giant reached a total length (TL) of more than 16 m. One single tooth can exceed more than 168 mm of total height [14]. The diagnostic characters of *C. megalodon* teeth include: large size, triangular shape, fine serrations on the cutting edges, a convex lingual face, a slightly convex to flat labial face, and a large v-shaped neck [7]. Juvenile specimens of *C. megalodon* can have lateral cusplets [15], or not [22]. The size and shape of the teeth vary within the jaw: anterior teeth are large and symmetrical whereas the latero-posterior teeth are asymmetrical with slanted crowns. Moving antero-posteriorly through the jaw, there is a slight initial increase in size on either side of the mid-line, followed by a progressive decrease that continues to the last tooth, e.g. [25] (Figure S1). Fossil teeth of *C. megalodon* range in age from 17 to 2 Ma (middle Miocene to Pleistocene) and have a cosmopolitan distribution [7], [14], [16].

Of relevance of this study, two shark paleo-nursery areas have previously been proposed: the Paleocene Williamsburg Formation of South Carolina, based on the presence of juvenile teeth of four lamnoid taxa [26]; and the late Oligocene Chandler Bridge Formation of South Carolina, based on the abundance of juvenile *Carcharocles* teeth, accompanied by small odontocete and mysticete skulls, which are assumed to be their prey species [16]. However, neither of the collections from these two localities have been rigorously analyzed and thus the presence of paleo-nurseries remained anecdotal until the present report.

The presence of mammals as potential prey does not represent evidence of a shark nursery area. As known from modern studies of sharks, the main purpose of the nursery areas is not feeding [1]–[4]. Studies have shown that some shark species do not consume large quantities of food during their juvenile stages [27]–[28]. Even when high-productivity nursery areas provide ample food resources for juvenile sharks, some species select these habitats more for predator avoidance and not food consumption [3]–[4]. Furthermore, some shark species present an ontogenetic shift in feeding patterns [29]–[32]. For example, the lamnoid white shark (*C. carcharias*) feeds mostly on fishes (including other sharks) during their juvenile stage and on mammals during their adult stage [33]–[35]. Marine mammals are not commonly found in the Gatun Formation. On the other hand, bony fish otoliths [36] and other shark species [7] are abundant, representing a food source for the marine fauna that lived in this diverse environment.

**Figure 2:**
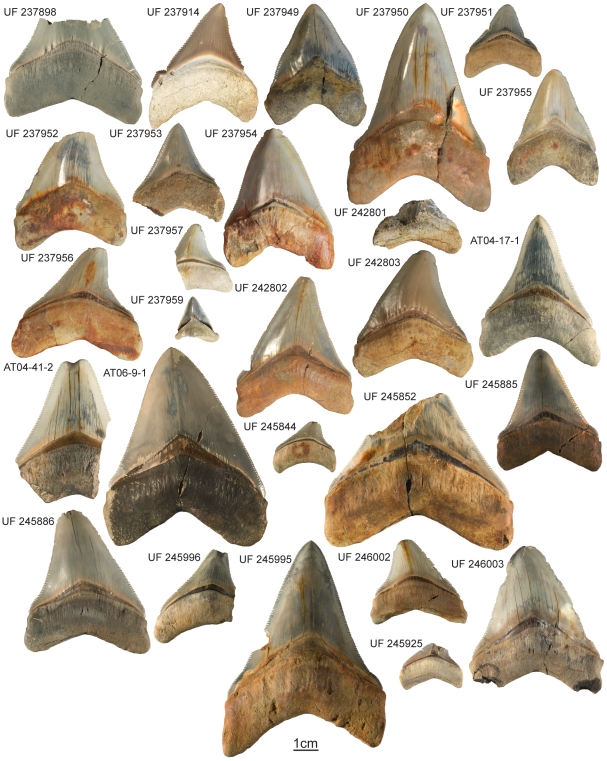
*Carcharocles megalodon* collection from the Gatun Formation. Specimens and their respective collection numbers. One specimen (CTPA 6671) was not available to photograph.

In this study *C. megalodon* teeth were collected and measured from two localities within the Gatun Formation of Panama (Figure 1). Surprisingly, large teeth are uncommon with specimens recovered having CH ranging between 16 to 72 mm (Figure 2). The objective of this work is to determine if the late Miocene Gatun Formation was used as a nursery area by young *C. megalodon*. Accordingly, we compared the tooth sizes from the Gatun Formation with those found in older and younger formations to determine if the smaller size distribution observed is unique to the species during the late Miocene. In addition, we compared these sizes with tooth sets from individuals of different life stages to determine if the small size observed is related to age, or position within the jaw. Finally, we calculated the TL of all *C. megalodon* individuals from the Gatun Formation to estimate their life stage. The results obtained in this study from tooth measurement comparisons and individual total length estimates allowed us to determine the age class/size of individuals that inhabited the shallow-water habitats of the late Miocene Gatun Formation, ∼10 million years ago.

## Results and Discussion

### Temporal comparisons of similar faunas

In many clades represented in the fossil record, animals oftentimes show a general tendency to become larger through time, i.e., also called “Cope's Rule” [37]–[38]. For example, within lamnoid sharks there is a chronoclinal trend towards increasing size of species within the genus *Carcharocles* from *Carcharocles auriculatus* to *Carcharocles angustidens* to its larger species, *Carcharocles megalodon* [16]. However, there is no evidence of such a microevolutionary trend within the single species *C. megalodon* through time, as we will show below.

**Figure 3:**
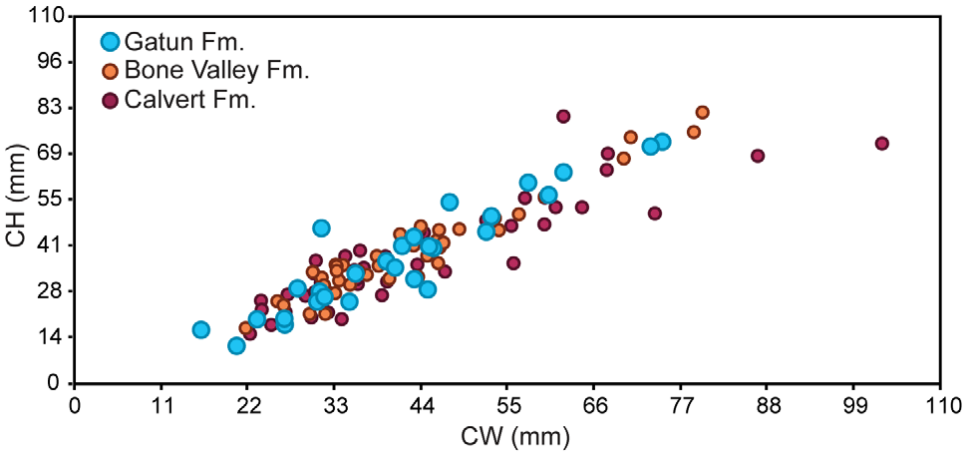
Temporal comparisons of similar faunas. Comparisons of *Carcharocles megalodon* tooth measurements (CH: crown height, CW: crown width), in millimeters from the Gatun Formation (late Miocene), with isolated teeth from a younger (Bone Valley, early Pliocene) and an older formation (Calvert, middle Miocene), which represent three localities from which this species is relatively abundant.

In order to know if the small size observed in the fossil *C. megalodon* from the Gatun Formation is a special feature during the late Miocene in a potentially chronoclinally evolving species, we performed tooth size comparisons through time within other marine faunas that have sufficiently large numbers of specimens of *C. megalodon*. Given the fact that the *C. megalodon* from the Calvert Formation of Maryland are older (∼14 Ma) and the *C. megalodon* from the Bone Valley Formation of Florida are younger (∼5 Ma), comparing these populations with *C. megalodon* from the Gatun Formation can determine if there is a long-term, chronoclinal trend for size increase, or if *C. megalodon* from the Gatun Formation are unusually small. Figure 3 shows that both large and small tooth sizes are found in the faunas older and younger than the Gatun Formation, and thus there is no observed microevolutionary trend for increased size in *C. megalodon* over time. We therefore assert that the small size observed in the Gatun Formation is not related to microevolutionary shifts in body size. Consequently, we demonstrate stasis in body size within the species *C. megalodon*, which provides us important context in which to compare ancient populations from the localities described above.

### Life stage comparisons

**Figure 4:**
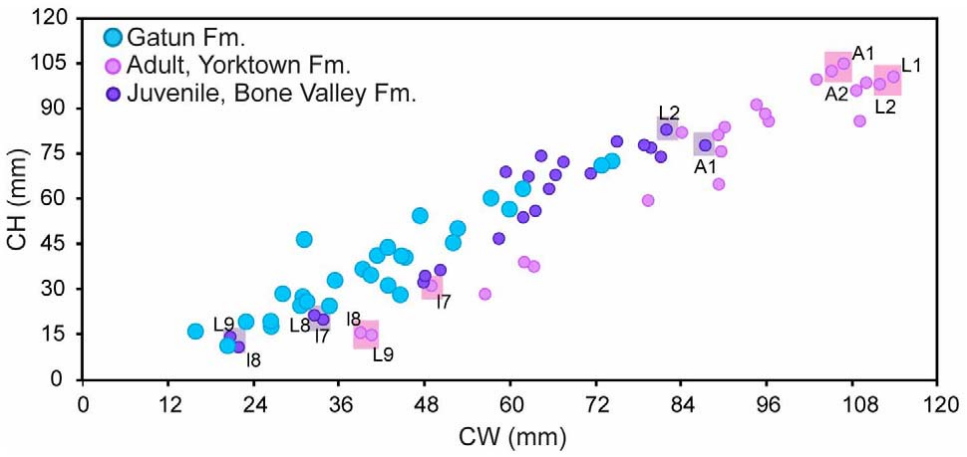
Life stage comparisons. Comparisons of *Carcharocles megalodon* tooth measurements (CH: crown height, CW: crown width) from the Gatun Formation with tooth sets of: a juvenile from the Bone Valley Formation and an adult from the Yorktown Formation. Note the size difference in relation with the tooth positions: larger teeth are the most anterior (e.g. A1, A2, L1, L2) whereas smaller teeth are the most lateral (e.g. L8, L9, l7, l8, l9). For more details on tooth positions, see figure S1.

It is known that within an individual, *C. megalodon* teeth vary in size within the jaw, e.g. [15]–[16], [25] (Figure S1). It could therefore be argued that the small size observed in the Gatun Formation is related to tooth position, rather than juvenile life stage of the individuals. In order to test this, we compared tooth sizes of the Gatun Formation specimens with associated tooth sets from individuals of different life stages (juvenile and adult) from other localities. Our results indicate that most teeth from the Gatun Formation are close to the observed range of a juvenile dentition (Figure 4), regardless of tooth position within the jaw.

Comparing the Gatun's isolated teeth with tooth sets of individuals from different life stages helps to determine if the tooth size observed is related with tooth position. Nevertheless, in order to determine the life stage of those animals were neonates, juveniles or adults; it is necessary to establish total length estimates as well, as presented below.

### Life stage and total length estimates

The tooth size comparisons made in this research suggest that the small size of *C. megalodon* teeth from the Gatun Formation is not related to temporal differences within a chronoclinally evolving species or to the tooth position within the jaw (as described above); but rather they belong to juvenile sharks. When only the teeth of a shark species are preserved, life (ontogenetic) stages of individuals can be predicted in two different ways: (1) studying morphological features of the teeth during juvenile stages; and (2) extrapolating total length using the relationship between body size and tooth crown height.

**Figure 5:**
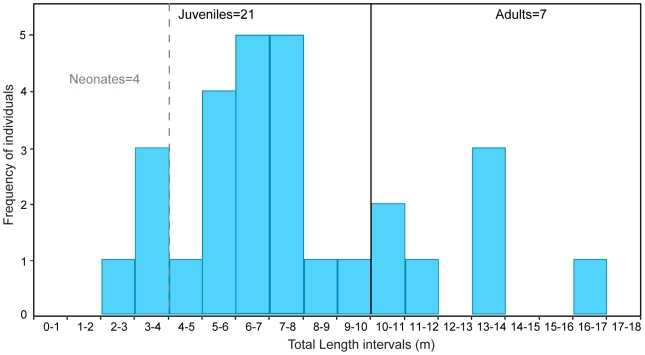
Total length histogram. Frequency of *Carcharocles megalodon* individuals at different life stages based on Gottfried et al. [14]. Neonates of *C. megalodon* reach until 4 m; juveniles until 10.5 m, and adults more than 10.5 m.

(1) In *C. megalodon*, teeth of juveniles sometimes demonstrate lateral cusplets [15] or not [22]. For example, UF 237914 (a lateral tooth) exhibits lateral cusplets and is assumed to be from a juvenile. On the other hand, UF 237959 (a lower anterior tooth) and UF 237949 (an upper anterior) are both very small teeth that exhibit no lateral cusplets (Figure 2). The latter teeth are small thick, heart-shaped, and are considered to represent embryonic *C*. *megalodon* teeth (Hubbell teeth). These latter teeth retain the morphology of the species even at small sizes and do no demonstrate lateral cusplets [22].

(2) Gottfried et al. (1996) [14] made inferences about the skeletal anatomy of *C. megalodon* based on comparisons with ontogenetic trends in the white shark, *Carcharodon carcharias*. They deduced that a *C. megalodon* fetus could reach ∼4 m, juveniles ∼10.5 m, and adults more than 10.5 m (∼17 m). Based on crown heights (CH) and following the work of Shimada (2003) [39], we estimate the total lengths (TL) of *C. megalodon* specimens from the Gatun Formation (Table 1). Based on Gottfried et al.'s inferences, the total length estimates made in this research suggest that the *C. megalodon* specimens from the Gatun Formation represent mostly juveniles (21 individuals), with total lengths less than 10.5 m, while a few specimens (7 individuals) are interpreted as adults, with an estimated total lengths beyond 10.5 m (Figure 5).

**Table 1:** *Carcharocles megalodon* isolated teeth measurements from the Gatun Formation, Panama.

Specimen	CW (mm)	CH (mm)	Position**	TL (m)***
UF 237898	53.0	50.0*	A1–A2	5.9
UF 237914	31.4	46.4	L1–L5	8.0
UF 237949	35.7	32.9	A1–A2	3.9
UF 237950	47.7	54.2	a2	7.3
UF 237951	26.8	17.6	L1–L5	3.1
UF 237952	43.2	31.3	L1–L5	5.4
UF 237953	30.9	24.5	l1–l5	7.2
UF 237954	41.7	41.2	A1–A2	4.9
UF 237955	28.4	28.5	A1–A2	3.4
UF 237956	44.9	28.1	l4–l6	16.8
UF 237957	26.7*	19.4	L6–L9	13.8
UF 237959	16.1	16.0	a1–a2	2.2
UF 242801	31.2	27.5*	L1–L5, l1–l5	6.4
UF 242802	45.1	41.0	L1–L5	7.1
UF 242803	40.8	34.7	L1–L5	6.0
AT04-17-1	43.2	43.8	a1–12	6.2
AT04-41-2	60.3	56.4	A1–A2	6.7
AT06-9-1	57.7	60.1	A1–A2	7.1
UF 245844	20.6	11.2	l5–l7	10.0
UF 245852	73.2	70.9*	L2–L4	10.8
UF 245885	39.6	36.6	L1–L3	5.2
UF 245886	45.6	40.5	L1–L5	7.0
UF 245996	31.8*	25.9	l3–l6	13.1
UF 245995	62.2	63.2	a3	11.0
UF 246002	35.0	24.5	L7–L9	11.5
UF 246003	52.4	45.4	L1–L3	6.4
UF 245925	23.2	19.2*	L6–L9	13.7
CTPA 6671	74.7	72.3	A1–A2	8.6

*Incomplete specimens. Tooth crown width (CW) and crown height (CH) measurements predicted using the line equation: y = mx+b (see figure S2).

**Range of possible positions where every tooth could have belonged (see figure S1 for position details).

***Total Length (TL) estimated based on Shimada (2003) [39] (see table S1). The value presented was calculated from the average among the different positions where every tooth could have belonged.

There is some expectation to find adult individuals inside a paleo-nursery area, along with the juvenile sharks for two reasons: (1) sharks constantly produce and shed teeth [40], if gravid females lay their eggs or give birth in nursery areas, one would expect to find some larger teeth; and (2) while nursery areas do offer some protection from larger predators, they do not necessarily keep all large individuals out [3], in fact, nursery areas are productive environments where competition for food can be very high [4].

### Concluding Remarks: Nursery area hypothesis

As described earlier, in addition to nurseries, extant sharks utilize adult feeding and reproduction areas [1]. Based on what is known about aggregations of the analogous species, the white shark (*C. carcharias*), we have also considered the hypothesis that the late Miocene Gatun Formation was used as an aggregation site by *C. megalodon* for feeding and/or reproduction rather than as a nursery area. Following their oceanic migrations, *C. carcharias* individuals aggregate in the eastern Pacific (also called SOFA or Shark CafÈ) [41]–[42]. They seasonally return to this pelagic area that is suggested to be used for feeding/foraging and mating [41]–[43]. Furthermore, *C. carcharias* also aggregates in various coastal “hot spots” where they feed around pinniped colonies [41]–[47]. Nevertheless, based on the presence of neonates of *C. megalodon*, the generally high proportion of juvenile individuals, the estimated shallow depth of the Gatun Formation and the scarcity of large mammals, we reject the hypothesis of the Gatun as a primarily area for reproduction or feeding.

In this study we show that the abundance of small tooth size observed in *C. megalodon* specimens from the Gatun Formation is not related to its temporal position within a chronoclinally evolving species or tooth position within the jaw. Thus, the *C. megalodon* from the Gatun Formation indicates the dominant juvenile life stage of individuals present from this fossil locality (with estimated body lengths between 2 and 10.5 meters). The *C. megalodon* and associated marine invertebrate and vertebrate faunas from the late Miocene Gatun Formation of Panama presents the typical characteristics of a shark nursery area, i.e., a shallow, productive environment that contains juveniles and neonates (the later indicating these individuals probably were born in the Gatun area). We therefore propose the Miocene Gatun Formation, as a nursery area that offered juvenile *C. megalodon* protection from larger predators and ample food resources (i.e. fishes).

Given that *C. megalodon* was the largest shark that has ever lived, it could be argued that this species may not have represented a potential prey for other sharks and therefore nursery areas would not be needed. In this study however, we report that neonate individuals of *C. megalodon* from the late Miocene Gatun Formation of Panama could be as small as 2 m long. Furthermore, many other shark species in the Gatun Formation apparently were sympatric with juvenile *C. megalodon*, including potential predators that can reach more than 6 m of total length (e.g. the great hammerhead shark (*Sphyrna mokarran*) and the extinct snaggletooth shark (*Hemipristis serra*)) [7]. Moreover, in spite of a juvenile dominance, adult individuals of *C. megalodon* (reaching until ∼17 m of TL) are also found in the Gatun Formation, representing additional potential predators. With regard to modern species, large-bodied sharks such as the tiger shark (*Galeocerdo cuvier*) and the great hammerhead (*S. mokarran*) also use nursery areas [48]. Additionally, it has been demonstrated that the modern apex shark predator of the oceans (and the analogous species of *C. megalodon* in this study), the white shark, uses the Southern California Bight as a nursery ground [49].

In summary, this study represents the first definitive evidence of an ancient shark nursery area from the Neotropics. Sharks are a very successful group that has been common in our oceans for at least 400 million years [40]. This research presents evidence that sharks have used nursery areas since ancient times, i.e., for at least 10 million years, and therefore extends the record of this behavior and adaptive strategy based on fossil evidence.

## Materials and Methods

*Carcharocles megalodon* teeth are relatively rare in the Gatun Formation. Of more than 400 teeth of fossil sharks collected from the Gatun Formation between 2007 and 2009 representing 16 taxa, a total of 28 specimens (Figure 2) of *C. megalodon* have been collected. Fossils do not provide a record of life as complete as when studying living organisms. For that reason and also because of the rarity of this species in the area of study, we consider our sample size adequate. In addition, it is urgent to study the fossils of a formation that will soon disappear due to the increasing excavations.

The two localities studied in the Neogene marine sediments of the Gatun Formation of Panama (Figure 1), crop out in a broad area in north-central Panama and have been proposed to be late Miocene, spanning from about 12 to 8.4 Ma [9]. The materials were collected mainly by surface prospecting by the Panama Canal Project Field Team of the Center of Tropical Paleobiology and Archaeology (CTPA) of the Smithsonian Tropical Research Institute (STRI), as well as the University of Florida (UF) scientists. Some of the specimens collected are deposited in the Florida Museum of Natural History (FLMNH) and are designated with a UF catalogue number which are available in its database: http://www.flmnh.ufl.edu/databases/VP/intro.htm. The remaining specimens are designated with a CTPA or AT number and are part of the STRI collection.

Crown height (CH) and width (CW) (Figure S2) of all specimens were measured (Table 1, S1, S2, S3 and S4) using digital calipers. In order to calculate dimensions of incomplete specimens, CW vs. CH data were plotted and a line regression was calculated (Figure S3). Measurements were then compared with isolated teeth from geologically younger and older collections and with different tooth sets from individuals of different life stages. The specimens' total lengths (TL) were calculated based on their CH.

### Temporal comparisons of similar faunas

Isolated teeth from the younger Bone Valley Formation, Florida (early Pliocene, ∼5 Ma) [50]–[51], from the Vertebrate Paleontology Collection at the FLMNH in Gainesville, Florida, were measured (Table S1) and compared with the Gatun teeth. Additionally, isolated teeth from the older Calvert Formation, Maryland (middle Miocene, ∼14 Ma) [52], from the Vertebrate Paleontology Collection at the U. S. National Museum of Natural History (NMNH), in Washington, D.C, were also measured (Table S2) and then compared with the Gatun Formation teeth.

### Life stage comparisons

Tooth sizes of the Gatun isolated teeth were measured and compared with two *C. megalodon* associated tooth sets of different life stages from the Hubbell collection at Gainesville, FL. The adult tooth set is from the Yorktown Formation, North Carolina (early Pliocene) [52] (Table S3). And the juvenile tooth set is from the Bone Valley Formation, Florida (early Pliocene) [50]–[51] (Table S4).

### Total length estimates

As described above, the extant white shark (*Carcharodon carcharias*), has been used as a general ecological analog to the extinct *Carcharocles megalodon*. Likewise, previous studies have asserted that teeth of *C. carcharias* can be used to estimate the total length of *C*. *megalodon* [14], [38]. Based on *C. carcharias* tooth height and total length ratios, we have measured *C. megalodon* tooth CH to extrapolate its TL estimates based on the work of Shimada (2003) [39] on the white shark, where every tooth position in the jaw corresponds to one regression equation that calculates its body size (Table S5). We assigned a range of possible positions to the Gatun teeth and estimated the TL of every specimen by calculating it from the average among the different positions where every tooth could have belonged (TL, Table 1). We then inferred the life stage of every *C. megalodon*, by extrapolating it from the relationship between body size and life stage in *C. carcharias* following Gottfried et al. (1996) [14]. We based our *C. megalodon* estimates on extrapolations from the extant *C. carcharias* given their similarities in body shape, feeding habits, and tooth and vertebral morphology. In addition, both species belong to the same order (Lamniformes), and in the absence of living members of the Otodontidae, *C*. *carcharias* is the most analogous species available.

## Supporting Information

Figure S1 Representation of a *Carcharocles megalodon* dentition. Tooth size and shape varies greatly within the jaw: most anterior teeth are larger and symmetrical; most lateral teeth are smaller and asymmetrical. Capital letters represent upper teeth, lowercase letters represent lower teeth. Letter A(a) is for anterior and L(l) for lateral. Adapted from Gottfried et al. (1996) [14].
(0.16 MB TIF)

Figure S2 Tooth measurement codes and dimensions. CW represents crown width and CH represents crown height. All measurements were taken in millimeters.
(0.07 MB TIF)

Figure S3 Tooth measurements line regressions. A. Known crown width (CW). Line regression calculated when is possible to measure the CW (i.e. CW in the x or independent axes) but the CH is unknown due to fossil preservation. B. Known crown height (CH). Line regression calculated when is possible to measure the CH (i.e. CH in the x or independent axes) but the CW is unknown due to fossil preservation.
(0.72 MB TIF)

Table S1 *Carcharocles megalodon* isolated teeth, from the Bone Valley Formation, Florida, USA.
(0.06 MB DOC)

Table S2 *Carcharocles megalodon* isolated teeth, from the Calvert Formation, Maryland, USA.
(0.07 MB DOC)

Table S3 Adult *Carcharocles megalodon* associated tooth set, from the Yorktown Formation, North Carolina, USA.
(0.04 MB DOC)

Table S4 Juvenile *Carcharocles megalodon* associated tooth set, from the Bone Valley Formation, Florida, USA.
(0.04 MB DOC)

Table S5 Total length regression based on CH of every tooth position, from Shimada (2003) [39].
(0.04 MB DOC)
