# Supplementary figures and images for: Ancient Nursery Area for the Extinct Giant Shark Megalodon from the Miocene of Panama
Source: PLoS One. 2010 May 10;5(5):e10552. doi: 10.1371/journal.pone.0010552 (PMC2866656; doi:10.1371/journal.pone.0010552)

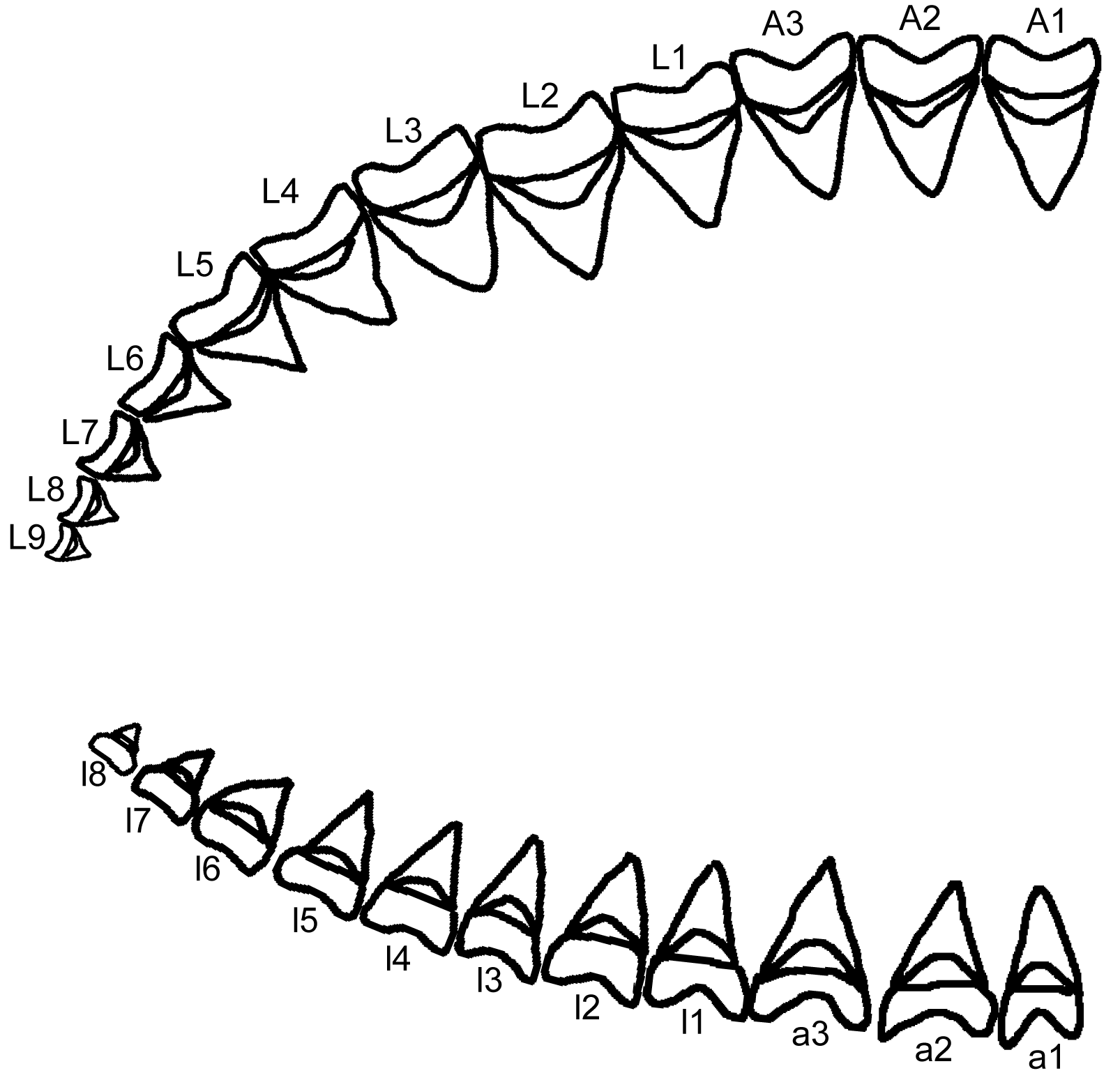

Supplement: Figure S1 — Representation of a Carcharocles megalodon dentition. Tooth size and shape varies greatly within the jaw: most anterior teeth are larger and symmetrical; most lateral teeth are smaller and asymmetrical. Capital letters represent upper teeth, lowercase letters represent lower teeth. Letter A(a) is for anterior and L(l) for lateral. Adapted from Gottfried et al. (1996) [14]. (0.16 MB TIF) [file pone.0010552.s001.tif]

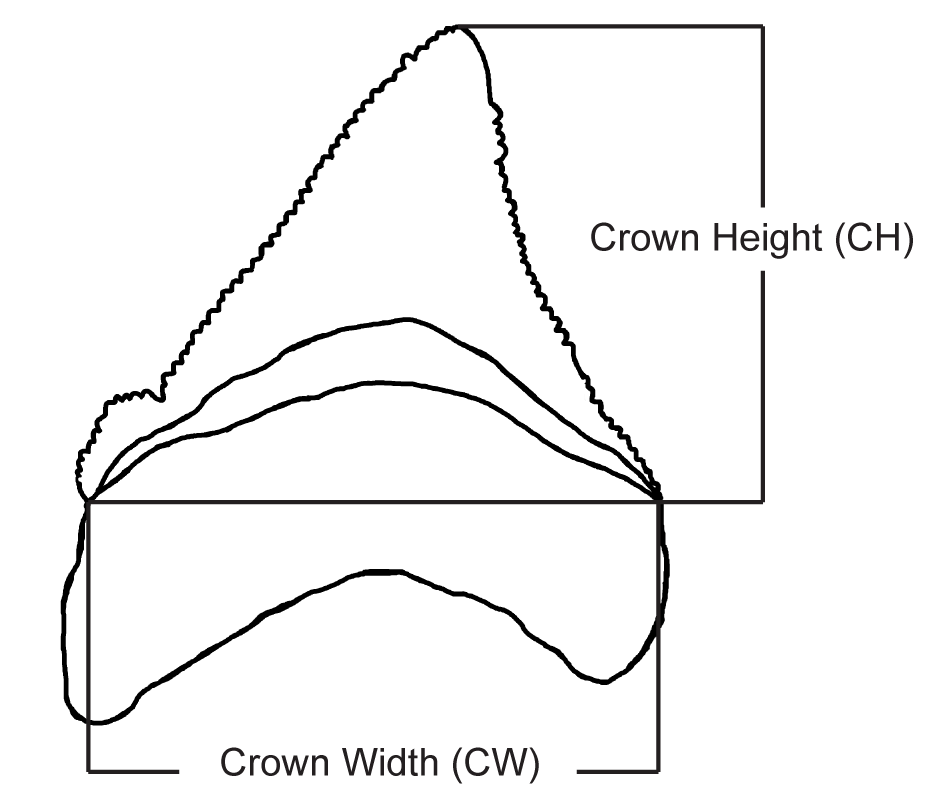

Supplement: Figure S2 — Tooth measurement codes and dimensions. CW represents crown width and CH represents crown height. All measurements were taken in millimeters. (0.07 MB TIF) [file pone.0010552.s002.tif]

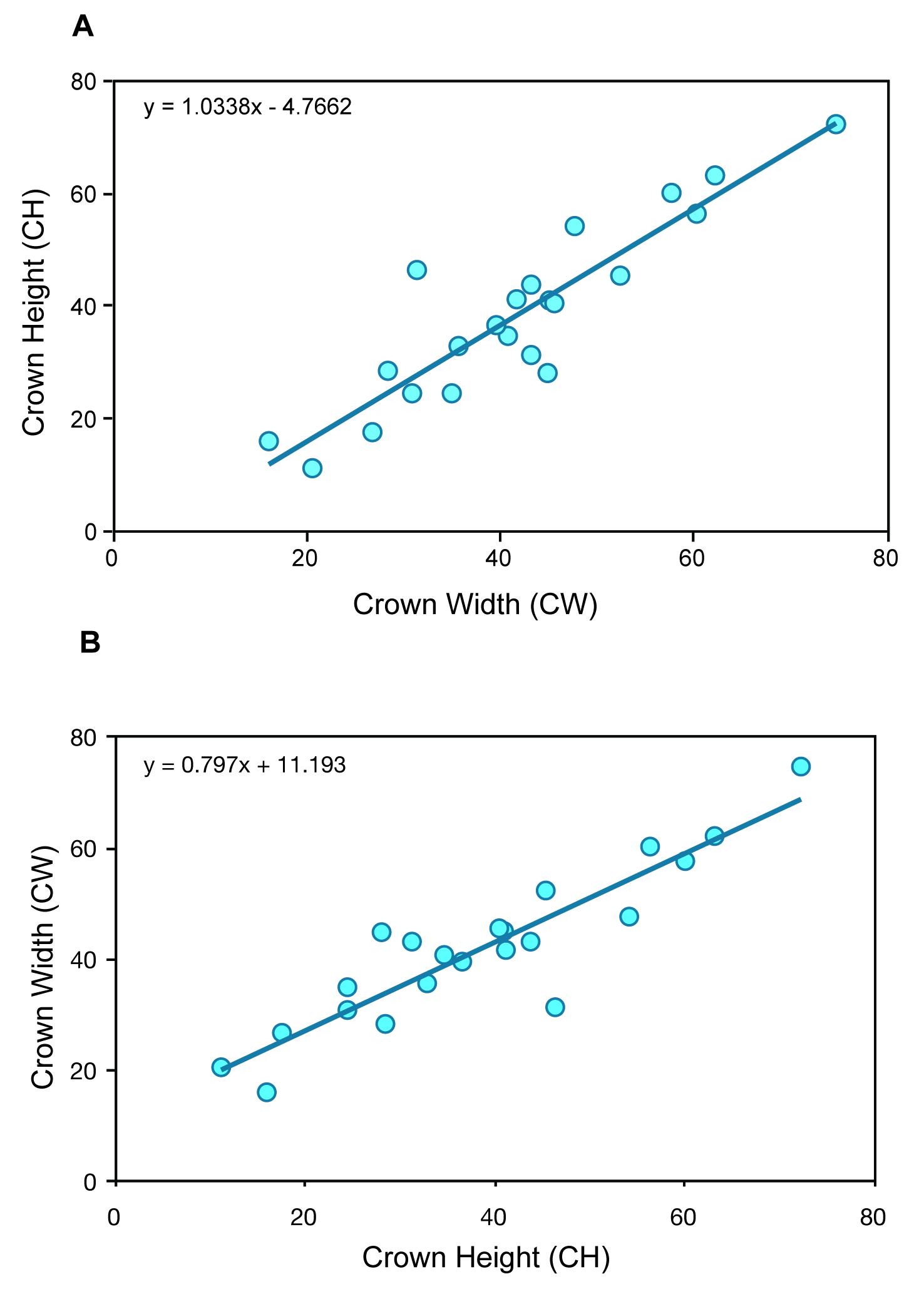

Supplement: Figure S3 — Tooth measurements line regressions. A. Known crown width (CW). Line regression calculated when is possible to measure the CW (i.e. CW in the x or independent axes) but the CH is unknown due to fossil preservation. B. Known crown height (CH). Line regression calculated when is possible to measure the CH (i.e. CH in the x or independent axes) but the CW is unknown due to fossil preservation. (0.72 MB TIF) [file pone.0010552.s003.tif]
