# Supplementary material for: Ancient Nursery Area for the Extinct Giant Shark Megalodon from the Miocene of Panama
Source: PLoS One. 2010 May 10;5(5):e10552. doi: 10.1371/journal.pone.0010552 (PMC2866656; doi:10.1371/journal.pone.0010552)
Supplement: Table S1 — Carcharocles megalodon isolated teeth, from the Bone Valley Formation, Florida, USA. (0.06 MB DOC) [file pone.0010552.s004.doc]

Table S1. C*archarocles megalodon* isolated teeth, from the Bone Valley Formation, Florida, USA.

| **Specimen** | **CW (mm)** | **CH (mm)** |
| --- | --- | --- |
| UF 217225 | 69.8 | 67.3 |
| UF 300 | 70.7 | 73.7 |
| UF 234583 | 79.8 | 81.1 |
| UF 217140 | 78.7 | 75.2 |
| UF 209170 | 59.8 | 55.8 |
| UF 228480 | 56.5 | 50.6 |
| UF 17850 | 53.4 | 49.4 |
| UF 17980 | 44.0 | 47.0 |
| UF 17850 | 48.9 | 46.1 |
| UF 228479 | 46.1 | 42.8 |
| UF 209164 | 46.4 | 40.7 |
| UF 17839 | 37.2 | 32.5 |
| UF 17839 | 33.7 | 30.8 |
| UF 24715 | 46.4 | 45.9 |
| UF 24715 | 41.4 | 44.6 |
| UF 24715 | 43.1 | 41.3 |
| UF 24715 | 38.4 | 38.2 |
| UF 24715 | 33.2 | 35.6 |
| UF 24715 | 43.7 | 31.9 |
| UF 17872 | 54.0 | 45.8 |
| UF 17872 | 44.9 | 38.3 |
| UF 17872 | 40.0 | 31.4 |
| UF 17872 | 36.2 | 33.6 |
| UF 17872 | 46.2 | 36.1 |
| UF 17872 | 34.1 | 35.4 |
| UF 17872 | 31.9 | 20.8 |
| UF 17872 | 25.8 | 24.6 |
| UF 17872 | 26.6 | 23.4 |
| UF 17872 | 31.5 | 31.7 |
| UF 55973 | 38.7 | 35.2 |
| UF 55973 | 35.1 | 29.6 |
| UF 17840 | 46.9 | 42.1 |
| UF 17840 | 33.2 | 27.0 |
| UF 17840 | 40.3 | 35.0 |
| UF 17840 | 21.8 | 16.6 |
| UF 132595 | 33.3 | 35.2 |
| UF 132588 | 31.7 | 29.4 |
| UF 132593 | 30.3 | 33.4 |
| UF 229807 | 33.4 | 33.7 |
