# Supplementary material for: Ancient Nursery Area for the Extinct Giant Shark Megalodon from the Miocene of Panama
Source: PLoS One. 2010 May 10;5(5):e10552. doi: 10.1371/journal.pone.0010552 (PMC2866656; doi:10.1371/journal.pone.0010552)
Supplement: Table S2 — Carcharocles megalodon isolated teeth, from the Calvert Formation, Maryland, USA. (0.07 MB DOC) [file pone.0010552.s005.doc]

Table S2. *Carcharocles megalodon* isolated teeth, from the Calvert Formation, Maryland, USA.

| **Specimen** | **CW (mm)** | **CH (mm)** |
| --- | --- | --- |
| USNM 457364 | 32.2 | 26.9 |
| DJB 2152 | 55.8 | 36.0 |
| USNM 494369 | 44.4 | 45.0 |
| USNM 489141 | 55.5 | 47.1 |
| USNM 475347 | 52.3 | 48.8 |
| USNM unnumbered | 67.7 | 63.9 |
| USNM 489136 | 86.8 | 68.1 |
| USNM 494370 | 102.6 | 71.8 |
| DJB 1029 | 27.1 | 26.6 |
| DJB 1566 | 29.4 | 26.2 |
| USNM 489137 | 43.9 | 45.6 |
| DJB 850 | 57.3 | 55.5 |
| DJB 1860 | 64.5 | 52.7 |
| DJB 1933 | 62.2 | 79.9 |
| DJB 1766 | 36.8 | 34.6 |
| DJB 1061 | 36.0 | 29.8 |
| DJB 1564 | 35.6 | 33.9 |
| ACC NO. 418873 | 30.1 | 19.8 |
| ACC NO. 413905 | 43.6 | 35.6 |
| DJB 1975 | 59.8 | 55.6 |
| DJB 934 | 26.9 | 21.4 |
| DJB 2009 | 25.0 | 17.5 |
| R.O. 411148 | 32.3 | 21.3 |
| USNM unnumbered | 47.1 | 33.5 |
| USNM 475303 | 34.4 | 38.1 |
| USNM 475306 | 39.8 | 30.5 |
| USNM 475299 | 30.4 | 33.0 |
| USNM unnumbered | 23.7 | 24.8 |
| USNM 475304 | 30.7 | 36.8 |
| USNM 473302 | 31.3 | 30.2 |
| USNM 475290 | 30.6 | 27.4 |
| USNM475297 | 34.0 | 19.3 |
| DJB 2090 | 67.8 | 68.8 |
| USNM4 95294 | 39.5 | 38.1 |
| PAL 535357 | 32.1 | 27.6 |
| USNM 26189 | 36.3 | 39.8 |
| USNM 171153 | 59.7 | 47.6 |
| USNM 171156 | 73.8 | 50.8 |
| USNM 24956 | 23.8 | 22.1 |
| USNM 24956 | 61.2 | 52.7 |
| USNM 171182 | 35.9 | 31.3 |
| USNM 337208 | 39.1 | 26.4 |
| USNM 171170 | 22.3 | 14.8 |
