# Supplementary material for: Ancient Nursery Area for the Extinct Giant Shark Megalodon from the Miocene of Panama
Source: PLoS One. 2010 May 10;5(5):e10552. doi: 10.1371/journal.pone.0010552 (PMC2866656; doi:10.1371/journal.pone.0010552)
Supplement: Table S3 — Adult Carcharocles megalodon associated tooth set, from the Yorktown Formation, North Carolina, USA. (0.04 MB DOC) [file pone.0010552.s006.doc]

Table S3. Adult *Carcharocles megalodon* associated tooth set, from the Yorktown Formation, North Carolina, USA.

| **Position*** | **CW (mm)** | **CH (mm)** |
| --- | --- | --- |
| A1 | 107.3 | 104.6 |
| A2 | 105.6 | 102.2 |
| A3 | 103.5 | 99.3 |
| L1 | 114.3 | 100.2 |
| L2 | 112.3 | 97.8 |
| L3 | 110.4 | 98.3 |
| L4 | 109.0 | 95.7 |
| L5 | 109.5 | 85.6 |
| L6 | 89.7 | 64.6 |
| L7 | 63.7 | 37.5 |
| L8 | 56.8 | 28.3 |
| L9 | 40.9 | 14.8 |
| a1 | 84.5 | 81.8 |
| a2 | 96.7 | 85.5 |
| a3 | 95.0 | 91.0 |
| l1 | 96.2 | 88.0 |
| l2 | 90.5 | 83.6 |
| l3 | 89.6 | 81.0 |
| l4 | 90.0 | 75.5 |
| l5 | 79.8 | 59.3 |
| l6 | 62.3 | 39.0 |
| l7 | 49.3 | 31.1 |
| l8 | 39.3 | 15.6 |

* For position details, see figure S1.
