# Supplementary material for: Ancient Nursery Area for the Extinct Giant Shark Megalodon from the Miocene of Panama
Source: PLoS One. 2010 May 10;5(5):e10552. doi: 10.1371/journal.pone.0010552 (PMC2866656; doi:10.1371/journal.pone.0010552)
Supplement: Table S4 — Juvenile Carcharocles megalodon associated tooth set, from the Bone Valley Formation, Florida, USA. (0.04 MB DOC) [file pone.0010552.s007.doc]

Table S4. Juvenile *Carcharocles megalodon* associated tooth set, from the Bone Valley Formation, Florida, USA.

| **Position*** | **CW (mm)** | **CH (mm)** |
| --- | --- | --- |
| A1 | 82.7 | 82.3 |
| A2 | 78.8 | 75.4 |
| A3 | 76.7 | 80.2 |
| L1 | 77.6 | 79.2 |
| L2 | 77.5 | 87.8 |
| L3 | 73.7 | 81.6 |
| L4 | 68.2 | 71.7 |
| L5 | 53.7 | 62.2 |
| L6 | 36.3 | 50.6 |
| L7 | 32.2 | 48.2 |
| L8 | 19.9 | 34.1 |
| L9 | 14.2 | 21.0 |
| a1 | 68.7 | 59.8 |
| a2 | 72.0 | 67.9 |
| a3 | 74.0 | 64.7 |
| l1 | 67.2 | 63.0 |
| l2 | 67.7 | 66.8 |
| l3 | 63.1 | 65.8 |
| l4 | 55.8 | 63.9 |
| l5 | 46.7 | 58.8 |
| l6 | 34.4 | 48.4 |
| l7 | 21.3 | 32.8 |
| l8 | 10.8 | 22.2 |

* For position details, see figure S1.
