# Supplementary material for: Ancient Nursery Area for the Extinct Giant Shark Megalodon from the Miocene of Panama
Source: PLoS One. 2010 May 10;5(5):e10552. doi: 10.1371/journal.pone.0010552 (PMC2866656; doi:10.1371/journal.pone.0010552)
Supplement: Table S5 — Total length regression based on CH of every tooth position, from Shimada (2003) [39]. (0.04 MB DOC) [file pone.0010552.s008.doc]

Table S5. Total length regression based on CH of every tooth position, Shimada [42].

| **Position** | **Regression Equation (x=CH)** |
| --- | --- |
| A1 | TL= 5.234+11.522x |
| A2 | TL= -2.16+12.103x |
| A3 | TL= 19.162+15.738x |
| L1 | TL= 5.540+14.197x |
| L2 | TL= 4.911+13.433x |
| L3 | TL= 0.464+14.550x |
| L4 | TL= 5.569+17.658x |
| L5 | TL= -5.778+26.381x |
| L6 | TL= -71.915+50.205x |
| L7 | TL= -48.696+69.292x |
| L8 | TL= -84.781+104.968x |
| L9 | TL= -10.765+17.616x |
| a1 | TL= -8.216+14.895x |
| a2 | TL= -7.643+13.597x |
| a3 | TL= -10.765+17.616x |
| l1 | TL= 9.962+17.437x |
| l2 | TL= 1.131+19.204x |
| l3 | TL= -30.947+25.132x |
| l4 | TL= -51.765+35.210x |
| l5 | TL= -73.120+55.262x |
| l6 | TL= -117.456+96.971x |
| l7 | TL= -64.732+138.350x |
| l8 | TL= -137.583+231.411x |
